# Supplementary material for: Modelling APOL1-mediated kidney inflammation and fibrosis using a partially reprogrammed urine-derived SIX2-positive renal progenitor cell line
Source: Stem Cell Res Ther. 2025 Nov 12;16:630. doi: 10.1186/s13287-025-04710-x (PMC12613565; doi:10.1186/s13287-025-04710-x)
Supplement: Supplementary file 4 — Supplementary material 4. [file 13287_2025_4710_MOESM4_ESM.pdf]

Supplementary Figure S1: Uncropped Western blots.

A (α-ACTININ 4)

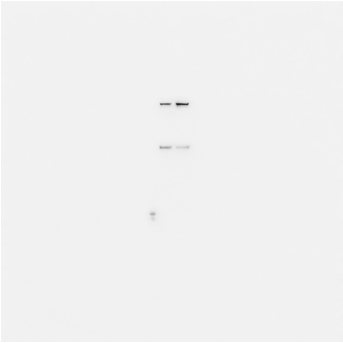

B (NPHS1)

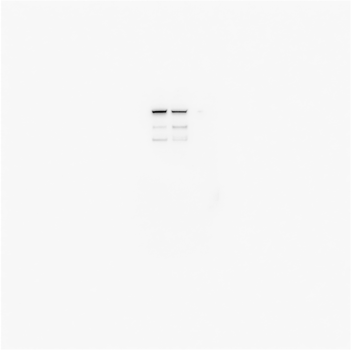

C (NPHS2)

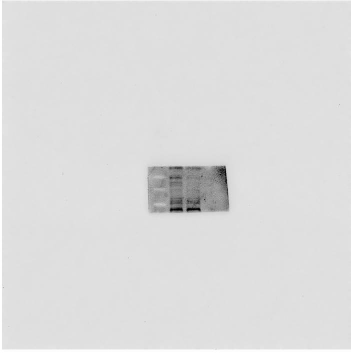

D (GAPDH)

E (Ladder)

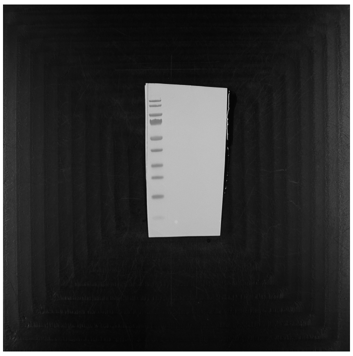

F (α-ACTININ 4)

G (NPHS1)

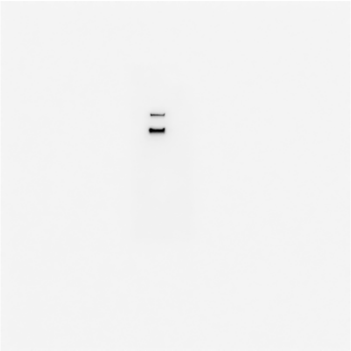

H (NPHS2)

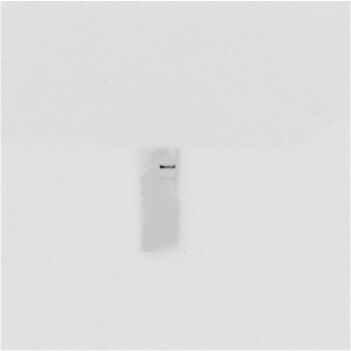

I (GAPDH)

J (Ladder)

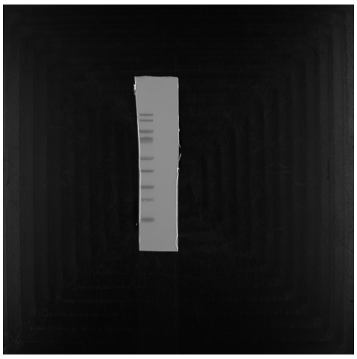

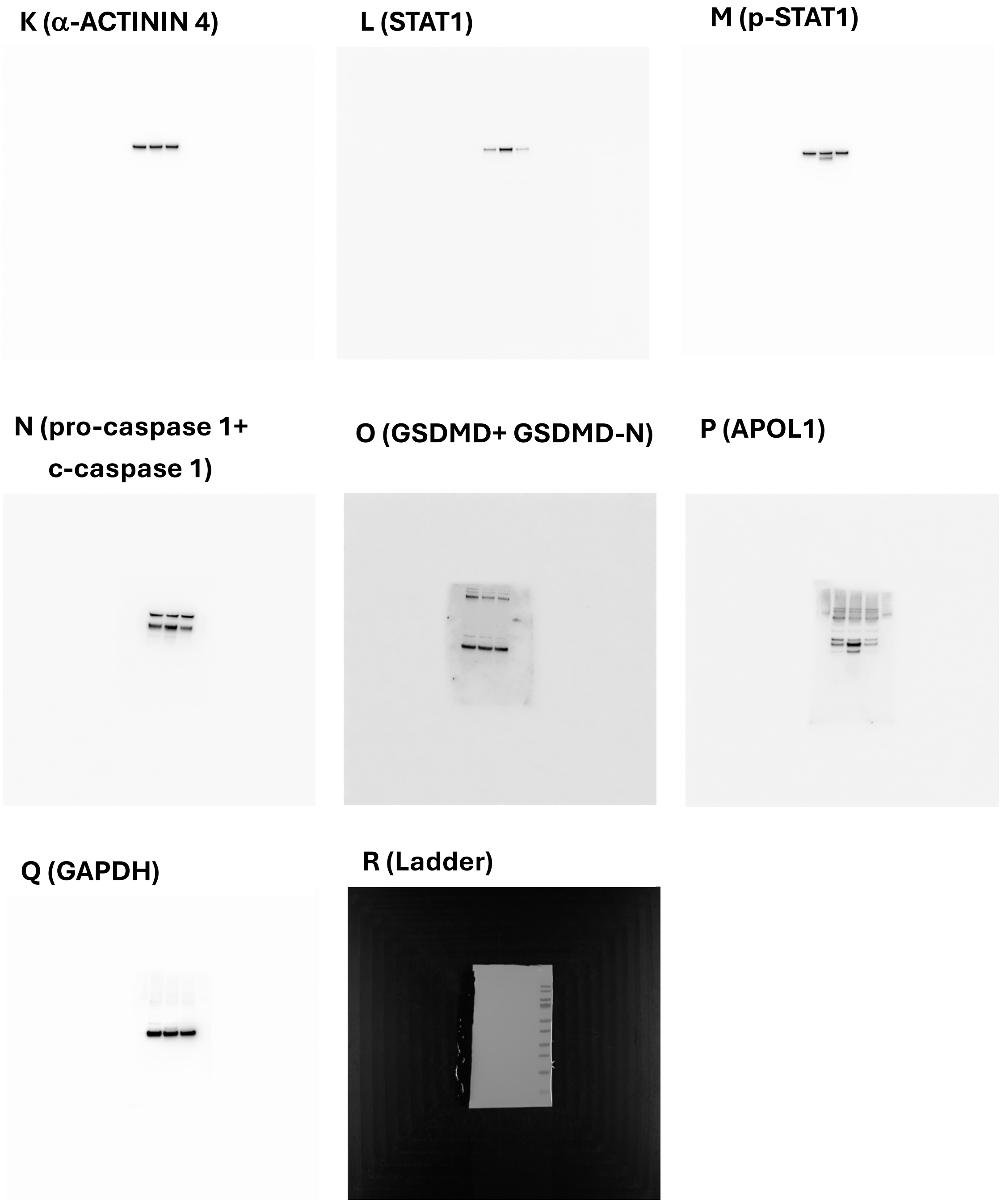

Supplementary Figure S1: Uncropped western blot images.
The detected proteins are presented from the left tot he right: A+F = $\alpha - \mathrm{ACTININ}\ 4$, B+G = NPHS1, C+H = NPHS2, D+I = GAPDH. Supplementary figure E+J represents the ladder fort he western blot images. The loading scheme starting next to the ladder from the right for the western blots A-D is as following: UM30-OSN and UM30-OSN podocyte. The loading scheme for the western blots F-I also starting next to the ladder has only one sample the human immortal podocyte line (AB 8/13). All proteins show the predicted kDA size. Figure K-P represents the uncropped blots for Figure 5. The loading scheme starting next to the ladder from the right like follows: UM30-OSN podocyte, UM30-OSN podocyte treated 24h with 100ng/ml IFN-γ and UM30-OSN podocyte treated with 48h 1µM Baricitinib and 24h 100ng/ml IFN-γ. For Western blots- K= α − ACTININ 4, L=STAT1, M= p-STAT1, N= pro-Caspase 1 and cleaved Caspase 1 (c-Caspase 1), O= Gasdermin D (GSDMD) and cleaved Gasdermin D (GSDMD-N), P= APOL1, Q= GAPDH and R=ladder. All proteins are detected under their corresponding size.
